# Dichloridobis(4-fluoro­aniline-κ*N*)zinc

**DOI:** 10.1107/S1600536812033922

**Published:** 2012-08-04

**Authors:** Ashokkumar Subashini, Kandasamy Ramamurthi, Helen Stoeckli-Evans

**Affiliations:** a Crystal Growth and Thin Film Laboratory, School of Physics, Bharathidasan University, Tiruchirappalli, Tamil Nadu 620 024, India; b Institute of Physics, University of Neuchâtel, Rue Emile-Argand 11, CH-2000 Neuchâtel, Switzerland

## Abstract

In the title compound, [ZnCl_2_(C_6_H_6_FN)_2_], the Zn^II^ atom has a slightly distorted tetra­hedral geometry, being coordinated by the N atoms of two 4-fluoro­aniline mol­ecules and the two Cl^−^ anions. The two benzene rings are almost perpendicular to one another, making a dihedral angle of 89.96 (13)°. In the crystal, mol­ecules are linked *via* pairs of N—H⋯Cl hydrogen bonds, forming chains propagating along the *b* axis. These chains are in turn linked *via* a second pair of N—H⋯Cl hydrogen bonds, forming a two-dimensional network parallel to the *ab* plane. The title compound crystallizes in the space group *Pca*2_1_ and exhibits weak second harmonic generation (SHG) properties.

## Related literature

For the measurement of second harmonic generation (SHG) conversion efficiency, see: Kurtz & Perry (1968 ▶). For the crystal structure of dichlorido-bis­(*p*-chloro­aniline)zinc, see: Subashini *et al.* (2012*a* ▶) and for the crystal structure of dichlorodo-bis­(*p*-bromo­aniline)zinc, see: Subashini *et al.* (2012*b* ▶); Feng *et al.* (2003 ▶).
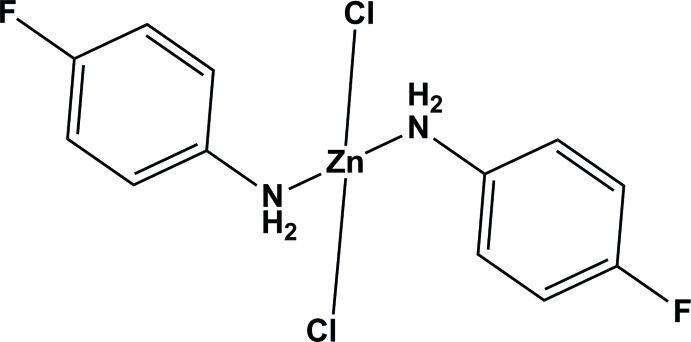


## Experimental

### 

#### Crystal data

[ZnCl_2_(C_6_H_6_FN)_2_]
*M* *_r_* = 358.51
Orthorhombic, 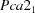

*a* = 11.6817 (5) Å
*b* = 4.7080 (2) Å
*c* = 25.2056 (15) Å
*V* = 1386.24 (12) Å^3^
*Z* = 4
Mo *K*α radiation
μ = 2.17 mm^−1^
*T* = 173 K
0.45 × 0.22 × 0.10 mm

#### Data collection

Stoe IPDS 2 diffractometer
Absorption correction: multi-scan (*MULscanABS* in *PLATON*; Spek, 2009 ▶) *T*_min_ = 0.742, *T*_max_ = 0.805
7963 measured reflections
2613 independent reflections
2465 reflections with *I* > 2σ(*I*)
*R*_int_ = 0.031

#### Refinement

*R*[*F* ^2^ > 2σ(*F* ^2^)] = 0.020
*wR*(*F* ^2^) = 0.043
*S* = 1.02
2613 reflections
172 parameters
1 restraint
H-atom parameters constrained
Δρ_max_ = 0.21 e Å^−3^
Δρ_min_ = −0.32 e Å^−3^
Absolute structure: Flack (1983 ▶), 1273 Friedel pairs
Flack parameter: 0.013 (10)

### 

Data collection: *X-AREA* (Stoe & Cie, 2009 ▶); cell refinement: *X-AREA*; data reduction: *X-RED32* (Stoe & Cie, 2009 ▶); program(s) used to solve structure: *SHELXS97* (Sheldrick, 2008 ▶); program(s) used to refine structure: *SHELXL97* (Sheldrick, 2008 ▶); molecular graphics: *PLATON* (Spek, 2009 ▶) and *Mercury* (Macrae *et al.*, 2008 ▶); software used to prepare material for publication: *SHELXL97*, *PLATON* and *publCIF* (Westrip, 2010 ▶).

## Supplementary Material

Crystal structure: contains datablock(s) I, global. DOI: 10.1107/S1600536812033922/is5178sup1.cif

Structure factors: contains datablock(s) I. DOI: 10.1107/S1600536812033922/is5178Isup2.hkl

Additional supplementary materials:  crystallographic information; 3D view; checkCIF report

## Figures and Tables

**Table 1:** Hydrogen-bond geometry (Å, °)

*D*—H⋯*A*	*D*—H	H⋯*A*	*D*⋯*A*	*D*—H⋯*A*
N1—H1*B*⋯Cl2^i^	0.92	2.63	3.436 (2)	147
N2—H2*B*⋯Cl1^i^	0.92	2.55	3.380 (2)	151
N1—H1*A*⋯Cl2^ii^	0.92	2.59	3.479 (2)	162
N2—H2*A*⋯Cl1^iii^	0.92	2.55	3.439 (2)	162

Symmetry codes: (i) 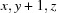; (ii) 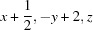; (iii) 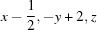.

**Fig. 1.**
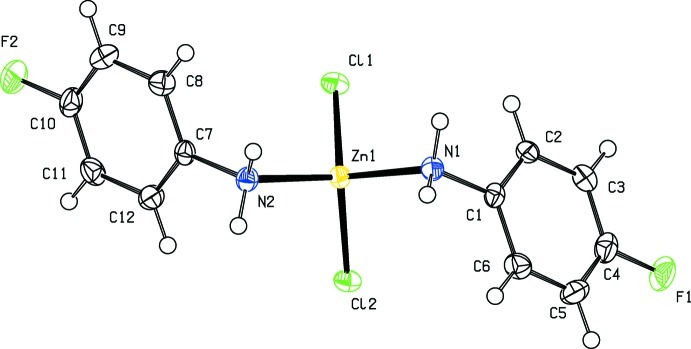
A view of the molecular structure of the title compound with the atom numbering. The displacement ellipsoids are drawn at the 50% probability level.

**Fig. 2.**
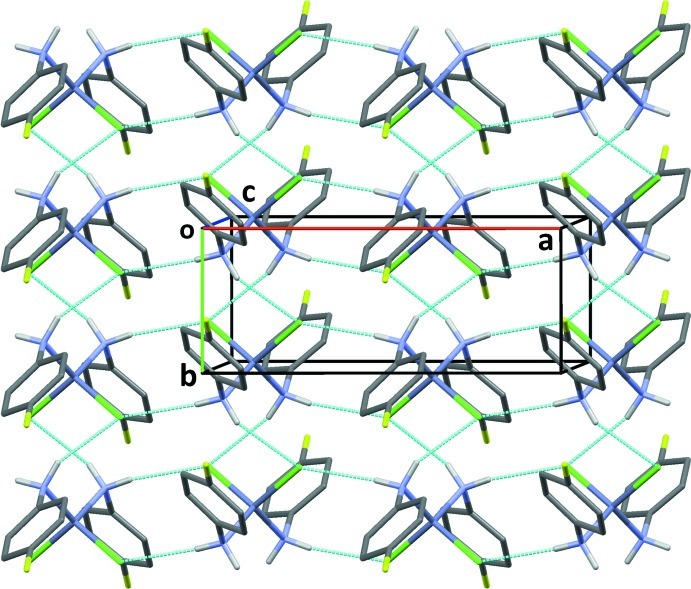
A view along the c axis of the crystal packing of the title compound. The N—H···Cl hydrogen bonds are shown as dashed cyan lines (see Table 1 for details).

## supplementary crystallographic information

### Comment

In our search for compounds exhibiting second harmonic generation (SHG) properties we have synthesized a series of ZnCl_2_ complexes of *p*-halogen substituted anilines. The title compound, the ZnCl_2_ complex of *p*-fluoroaniline crystallized in the noncentrosymmetric orthorhombic space group Pca2_1_, while the *p*-chloroaniline (Subashini *et al.*, 2012*a*) and *p*-bromoaniline (Subashini *et al.*, 2012*b*; Feng *et al.*, 2003) ZnCl_2_ complexes crystallized in the centrosymmetric monoclinic space group *C*2/*c* and both molecules have crystallographic 2-fold rotation symmetry.

In the title compound (Fig. 1), the zinc atom has a slightly distorted tetrahedral geometry, being coordinated by the atoms N1 and N2 of two *p*-fluoroaniline molecules and the two Cl^-^ anions. The two benzene rings (C1—C6 and C7—C12) are perpendicular to one another with a dihedral angle of 89.96 (13)°. In the *p*-chloroaniline and *p*-bromoaniline ZnCl_2_ complexes mentioned above the same angles are 80.65 (16) and 80.0 (3)°, respectively.

In the crystal of the title compound, molecules are linked *via* a pair of N—H···Cl hydrogen bonds forming chains propagating along the *b* axis direction. These chains are in turn linked *via* a second pair of N—H···Cl hydrogen bonds to form a two-dimensional network lying parallel to the *ab* plane (Table 1 and Fig. 2). This contrasts with the packing in the crystals of the *p*-chloroaniline and *p*-bromoaniline ZnCl_2_ complexes. There molecules are linked by four N—H···halogen bonds to form chains propagating along [010], with no significant interactions between the chains.

As the title compound crystallized in a noncentrosymmetric space group it was decided to measure the second harmonic generation (SHG) properties of all three compounds; dichloro-bis(*p*-fluoroaniline)zinc, dichloro-bis(*p*-chloroaniline)zinc and dichloro-bis(*p*-bromoaniline)zinc. The SHG conversion efficiency was determined by the powder technique developed by (Kurtz & Perry, 1968). The crystals were powdered and the fine powdered samples were inserted in a micro-capillary tube and then subjected to a Q-switched Nd: YAG laser emitting 1064 nm radiation with 3.9 mJ/pulse. The frequency doubling was confirmed by the emission of green radiation of wavelength 532 nm collected by a monochromator after separating the 1064 nm pump beam with an IR-blocking filter. A detector connected to a power meter was used to detect the second harmonic intensity.

The output beam voltage produced by dichloro-bis(*p*-fluoroaniline)zinc, dichloro-bis(*p*-chloroaniline)zinc and dichloro-bis(*p*-bromoaniline)zinc derivatives were 15, 3 and 10 mV, respectively. The same quantity of crystalline KDP (potassium dihydrogen phosphate) powder, used as a reference material, produced 140 mV as output beam voltage. Hence the three samples exhibits SHG efficiency of only *ca* 0.11, 0.02 and 0.07 times that of the KDP.

### Experimental

The title compound was prepared by the condensation reaction of *p*-fluoroaniline with ZnCl_2_ in a 1:1 molar ratio. The reaction mixture was dissolved in methanol and heated under reflux for 6 h. The resulting solution was filtered and allowed to evaporate. Colourless rod-like crystals of the title compound, suitable for X-ray diffraction analysis, were obtained in a period of *ca* 7 days. The same method was used for the preparation of the *p*-chloroaniline and *p*-bromoaniline ZnCl_2_ complexes.

### Refinement

All the H atoms could be located in a difference Fourier map. In the final cycles of refinement they were included in calculated positions and treated as riding atoms: N—H = 0.92 Å and C—H = 0.95 Å with *U*_iso_(H) = 1.2*U*_eq_(N or C).

### Crystal data

**Table:**

[ZnCl_2_(C_6_H_6_FN)_2_]	*F*(000) = 720
*M**_r_* = 358.51	*D*_x_ = 1.718 Mg m^−^^3^
Orthorhombic, *P**c**a*2_1_	Mo *K*α radiation, λ = 0.71073 Å
Hall symbol: P 2c -2ac	Cell parameters from 10039 reflections
*a* = 11.6817 (5) Å	θ = 1.6–26.1°
*b* = 4.7080 (2) Å	µ = 2.17 mm^−^^1^
*c* = 25.2056 (15) Å	*T* = 173 K
*V* = 1386.24 (12) Å^3^	Rod, colourless
*Z* = 4	0.45 × 0.22 × 0.10 mm

### Data collection

**Table:**

Stoe IPDS 2 diffractometer	2613 independent reflections
Radiation source: fine-focus sealed tube	2465 reflections with *I* > 2σ(*I*)
Plane graphite monochromator	*R*_int_ = 0.031
φ and ω scans	θ_max_ = 25.6°, θ_min_ = 1.6°
Absorption correction: multi-scan (MULscanABS in *PLATON*; Spek, 2009)	*h* = −14→12
*T*_min_ = 0.742, *T*_max_ = 0.805	*k* = −5→5
7963 measured reflections	*l* = −30→30

### Refinement

**Table:**

Refinement on *F*^2^	Secondary atom site location: difference Fourier map
Least-squares matrix: full	Hydrogen site location: inferred from neighbouring sites
*R*[*F*^2^ > 2σ(*F*^2^)] = 0.020	H-atom parameters constrained
*wR*(*F*^2^) = 0.043	*w* = 1/[σ^2^(*F*_o_^2^) + (0.0253*P*)^2^] where *P* = (*F*_o_^2^ + 2*F*_c_^2^)/3
*S* = 1.02	(Δ/σ)_max_ = 0.001
2613 reflections	Δρ_max_ = 0.21 e Å^−^^3^
172 parameters	Δρ_min_ = −0.32 e Å^−^^3^
1 restraint	Absolute structure: Flack (1983), 1273 Friedel pairs
Primary atom site location: structure-invariant direct methods	Flack parameter: 0.013 (10)

### Special details

**Table:**

Geometry. Bond distances, angles *etc*. have been calculated using the rounded fractional coordinates. All su's are estimated from the variances of the (full) variance-covariance matrix. The cell e.s.d.'s are taken into account in the estimation of distances, angles and torsion angles

### Fractional atomic coordinates and isotropic or equivalent isotropic displacement parameters (Å^2^)

**Table:**

	*x*	*y*	*z*	*U*_iso_*/*U*_eq_	
Zn1	0.11820 (2)	0.94853 (5)	0.29991 (2)	0.0175 (1)	
Cl1	0.24924 (5)	0.67311 (13)	0.25893 (2)	0.0231 (2)	
Cl2	−0.01369 (5)	0.66951 (13)	0.33956 (2)	0.0224 (2)	
F1	0.24874 (17)	0.4882 (4)	0.53656 (6)	0.0375 (5)	
F2	0.00539 (17)	0.6379 (4)	0.05020 (6)	0.0386 (6)	
N1	0.19821 (18)	1.1742 (4)	0.35892 (7)	0.0190 (6)	
N2	0.03682 (17)	1.1921 (5)	0.24427 (7)	0.0193 (6)	
C1	0.2140 (2)	1.0033 (5)	0.40640 (10)	0.0173 (8)	
C2	0.3039 (2)	0.8160 (6)	0.40935 (9)	0.0206 (8)	
C3	0.3164 (2)	0.6390 (6)	0.45336 (9)	0.0236 (8)	
C4	0.2366 (2)	0.6601 (6)	0.49308 (9)	0.0267 (8)	
C5	0.1466 (2)	0.8448 (7)	0.49146 (10)	0.0279 (8)	
C6	0.1343 (2)	1.0192 (6)	0.44767 (12)	0.0247 (9)	
C7	0.0273 (2)	1.0505 (5)	0.19283 (10)	0.0181 (7)	
C8	0.1041 (2)	1.1133 (7)	0.15318 (10)	0.0242 (9)	
C9	0.0976 (3)	0.9741 (7)	0.10479 (11)	0.0305 (9)	
C10	0.0127 (2)	0.7762 (6)	0.09785 (9)	0.0263 (8)	
C11	−0.0645 (2)	0.7084 (6)	0.13658 (10)	0.0258 (8)	
C12	−0.0570 (2)	0.8484 (6)	0.18522 (9)	0.0236 (8)	
H1A	0.26840	1.23560	0.34700	0.0230*	
H1B	0.15510	1.33170	0.36710	0.0230*	
H2	0.35790	0.80690	0.38120	0.0250*	
H2A	−0.03540	1.23620	0.25630	0.0230*	
H2B	0.07640	1.35950	0.24000	0.0230*	
H3	0.37820	0.50830	0.45570	0.0280*	
H5	0.09330	0.85340	0.51990	0.0340*	
H6	0.07220	1.14880	0.44570	0.0300*	
H8	0.16170	1.25240	0.15890	0.0290*	
H9	0.15060	1.01470	0.07720	0.0370*	
H11	−0.12190	0.56930	0.13050	0.0310*	
H12	−0.10940	0.80520	0.21290	0.0280*	

### Atomic displacement parameters (Å^2^)

**Table:**

	*U*^11^	*U*^22^	*U*^33^	*U*^12^	*U*^13^	*U*^23^
Zn1	0.0174 (1)	0.0186 (1)	0.0167 (1)	−0.0009 (1)	−0.0010 (1)	−0.0002 (2)
Cl1	0.0180 (3)	0.0236 (3)	0.0277 (3)	0.0019 (3)	0.0033 (2)	−0.0017 (2)
Cl2	0.0187 (3)	0.0224 (3)	0.0261 (3)	−0.0031 (3)	0.0034 (2)	−0.0004 (2)
F1	0.0460 (9)	0.0433 (11)	0.0232 (8)	−0.0064 (9)	−0.0048 (9)	0.0147 (6)
F2	0.0423 (10)	0.0516 (13)	0.0218 (7)	0.0015 (9)	−0.0016 (6)	−0.0145 (7)
N1	0.0200 (10)	0.0179 (11)	0.0191 (9)	−0.0029 (9)	−0.0014 (8)	0.0020 (8)
N2	0.0215 (11)	0.0185 (11)	0.0180 (10)	0.0010 (10)	−0.0011 (8)	−0.0016 (8)
C1	0.0214 (13)	0.0145 (15)	0.0161 (13)	−0.0048 (10)	−0.0028 (10)	−0.0017 (8)
C2	0.0168 (12)	0.0252 (15)	0.0199 (12)	−0.0052 (11)	−0.0001 (9)	−0.0016 (10)
C3	0.0199 (13)	0.0231 (14)	0.0277 (13)	0.0009 (11)	−0.0043 (10)	−0.0005 (10)
C4	0.0335 (15)	0.0261 (14)	0.0204 (12)	−0.0074 (13)	−0.0050 (10)	0.0044 (10)
C5	0.0295 (14)	0.0329 (17)	0.0214 (12)	−0.0033 (14)	0.0073 (11)	0.0003 (12)
C6	0.0221 (15)	0.0241 (17)	0.0279 (15)	0.0057 (13)	0.0006 (11)	−0.0025 (10)
C7	0.0200 (12)	0.0171 (12)	0.0172 (12)	0.0035 (11)	−0.0028 (10)	0.0009 (10)
C8	0.0232 (14)	0.0240 (17)	0.0254 (14)	−0.0010 (13)	0.0010 (10)	−0.0001 (11)
C9	0.0298 (15)	0.040 (2)	0.0216 (13)	−0.0025 (14)	0.0068 (11)	0.0018 (11)
C10	0.0305 (14)	0.0306 (15)	0.0179 (12)	0.0081 (13)	−0.0026 (10)	−0.0057 (11)
C11	0.0213 (12)	0.0277 (16)	0.0284 (14)	−0.0025 (12)	−0.0050 (10)	−0.0069 (11)
C12	0.0241 (13)	0.0254 (14)	0.0212 (13)	0.0008 (12)	0.0021 (10)	0.0005 (10)

### Geometric parameters (Å, º)

**Table:**

Zn1—Cl1	2.2565 (7)	C4—C5	1.365 (4)
Zn1—Cl2	2.2579 (7)	C5—C6	1.383 (4)
Zn1—N1	2.0530 (19)	C7—C8	1.375 (3)
Zn1—N2	2.046 (2)	C7—C12	1.383 (3)
F1—C4	1.370 (3)	C8—C9	1.387 (4)
F2—C10	1.369 (3)	C9—C10	1.372 (4)
N1—C1	1.454 (3)	C10—C11	1.367 (3)
N2—C7	1.462 (3)	C11—C12	1.395 (4)
N1—H1B	0.9200	C2—H2	0.9500
N1—H1A	0.9200	C3—H3	0.9500
N2—H2A	0.9200	C5—H5	0.9500
N2—H2B	0.9200	C6—H6	0.9500
C1—C6	1.398 (4)	C8—H8	0.9500
C1—C2	1.373 (3)	C9—H9	0.9500
C2—C3	1.395 (4)	C11—H11	0.9500
C3—C4	1.372 (3)	C12—H12	0.9500
			
Cl1—Zn1—Cl2	109.34 (2)	C1—C6—C5	119.5 (2)
Cl1—Zn1—N1	108.68 (6)	N2—C7—C12	119.4 (2)
Cl1—Zn1—N2	108.88 (6)	N2—C7—C8	119.8 (2)
Cl2—Zn1—N1	106.92 (6)	C8—C7—C12	120.8 (2)
Cl2—Zn1—N2	108.21 (6)	C7—C8—C9	120.1 (3)
N1—Zn1—N2	114.72 (8)	C8—C9—C10	118.2 (3)
Zn1—N1—C1	111.58 (14)	F2—C10—C11	118.3 (2)
Zn1—N2—C7	112.81 (16)	F2—C10—C9	118.7 (2)
C1—N1—H1A	109.00	C9—C10—C11	123.0 (2)
C1—N1—H1B	109.00	C10—C11—C12	118.4 (2)
H1A—N1—H1B	108.00	C7—C12—C11	119.5 (2)
Zn1—N1—H1B	109.00	C1—C2—H2	120.00
Zn1—N1—H1A	109.00	C3—C2—H2	120.00
Zn1—N2—H2B	109.00	C2—C3—H3	121.00
Zn1—N2—H2A	109.00	C4—C3—H3	121.00
H2A—N2—H2B	108.00	C4—C5—H5	121.00
C7—N2—H2A	109.00	C6—C5—H5	121.00
C7—N2—H2B	109.00	C1—C6—H6	120.00
N1—C1—C6	119.9 (2)	C5—C6—H6	120.00
N1—C1—C2	119.8 (2)	C7—C8—H8	120.00
C2—C1—C6	120.2 (2)	C9—C8—H8	120.00
C1—C2—C3	120.4 (2)	C8—C9—H9	121.00
C2—C3—C4	117.8 (2)	C10—C9—H9	121.00
C3—C4—C5	123.2 (2)	C10—C11—H11	121.00
F1—C4—C3	118.1 (2)	C12—C11—H11	121.00
F1—C4—C5	118.7 (2)	C7—C12—H12	120.00
C4—C5—C6	118.8 (2)	C11—C12—H12	120.00
			
Cl1—Zn1—N1—C1	−80.53 (15)	C2—C3—C4—F1	−179.6 (2)
Cl2—Zn1—N1—C1	37.40 (16)	C2—C3—C4—C5	−0.1 (4)
N2—Zn1—N1—C1	157.37 (14)	F1—C4—C5—C6	179.8 (2)
Cl1—Zn1—N2—C7	31.74 (16)	C3—C4—C5—C6	0.3 (4)
Cl2—Zn1—N2—C7	−87.00 (15)	C4—C5—C6—C1	−0.2 (4)
N1—Zn1—N2—C7	153.74 (15)	N2—C7—C8—C9	178.2 (3)
Zn1—N1—C1—C2	80.9 (2)	C12—C7—C8—C9	0.1 (4)
Zn1—N1—C1—C6	−95.7 (2)	N2—C7—C12—C11	−178.5 (2)
Zn1—N2—C7—C8	−98.8 (2)	C8—C7—C12—C11	−0.5 (4)
Zn1—N2—C7—C12	79.2 (2)	C7—C8—C9—C10	0.5 (4)
N1—C1—C2—C3	−176.3 (2)	C8—C9—C10—F2	179.9 (2)
C6—C1—C2—C3	0.3 (4)	C8—C9—C10—C11	−0.8 (5)
N1—C1—C6—C5	176.6 (2)	F2—C10—C11—C12	179.8 (2)
C2—C1—C6—C5	−0.1 (4)	C9—C10—C11—C12	0.5 (4)
C1—C2—C3—C4	−0.2 (4)	C10—C11—C12—C7	0.2 (4)

### Hydrogen-bond geometry (Å, º)

**Table:**

*D*—H···*A*	*D*—H	H···*A*	*D*···*A*	*D*—H···*A*
N1—H1*B*···Cl2^i^	0.92	2.63	3.436 (2)	147
N2—H2*B*···Cl1^i^	0.92	2.55	3.380 (2)	151
N1—H1*A*···Cl2^ii^	0.92	2.59	3.479 (2)	162
N2—H2*A*···Cl1^iii^	0.92	2.55	3.439 (2)	162

Symmetry codes: (i) *x*, *y*+1, *z*; (ii) *x*+1/2, −*y*+2, *z*; (iii) *x*−1/2, −*y*+2, *z*.
